# Trefoil factor 3 promotes pancreatic carcinoma progression via WNT pathway activation mediated by enhanced WNT ligand expression

**DOI:** 10.1038/s41419-022-04700-4

**Published:** 2022-03-25

**Authors:** Feifei Cheng, Xuejuan Wang, Yi-Shiou Chiou, Chuyu He, Hui Guo, Yan Qin Tan, Basappa Basappa, Tao Zhu, Vijay Pandey, Peter E. Lobie

**Affiliations:** 1 Tsinghua-Berkeley Shenzhen Institute and The Institute of Biopharmaceutical and Health Engineering Tsinghua Shenzhen International Graduate School, Tsinghua University, Shenzhen, 518055 People’s Republic of China; 2 Shenzhen Bay Laboratory, Shenzhen, 518055 People’s Republic of China; 3 Department of Studies in Organic Chemistry, University of Mysore, Mysore, 570005 India; 4 Department of Oncology of the First Affiliated Hospital of USTC, Division of Life Sciences and Medicine, University of Science and Technology of China, Anhui Hefei, 230027 People’s Republic of China

**Keywords:** Oncogenesis, Pancreatic cancer

## Abstract

Pancreatic ductal adenocarcinoma (PDAC) is a major cause of cancer-related mortality with a dismal prognosis that has changed little over the past few decades. Further understanding of the molecular pathology of PDAC progression is urgently required in order to improve the prognosis of patients with PDAC. Herein, it was observed that trefoil factor 3 (TFF3) expression was elevated in PDAC, and was positively correlated with a worse overall patient survival outcome. Forced expression of TFF3 promoted oncogenic functions of PDAC cells in vitro including cell proliferation, survival, foci formation, cancer stem cell-like behavior and invasion, ex vivo colony growth in 3D-Matrigel, and xenograft growth in vivo. Depletion or pharmacological inhibition of TFF3 inhibited these same processes. RNA-Seq analysis and subsequent mechanistic analyses demonstrated that TFF3 increased the expression of various WNT ligands to mediate WNT pathway activation required for TFF3-stimulated PDAC progression. Combined pharmacological inhibition of TFF3 and WNT signaling significantly attenuated PDAC xenograft growth and potentiated the therapeutic efficacy of gemcitabine in both ex vivo and in vivo models. Hence, a mechanistic basis for combined inhibition of pathways enhancing PDAC progression is provided and suggests that inhibition of TFF3 may assist to ameliorate outcomes in PDAC.

## Introduction

The overall prognosis of patients with pancreatic ductal adenocarcinoma (PDAC) remains poor, with a 5-year survival rate of 8% [1]. Although a number of discrete genetic alterations associated with PDAC development and progression have been identified [2, 3], potent and non-mutated oncogenic drivers [4] that contribute to PDAC progression are still not well determined. Characterization of such oncogenes is necessary for expanding therapeutic strategies and prolonging the poor survival in patients with PDAC.

The human trefoil factor 3 (*TFF3*) gene encodes a small secreted protein of 59 amino acids with a trefoil structure [5]. TFF3 is a promiscuous ligand that activates multiple signaling pathways, including EGFR, CXCR4/7, HER2, MET, SRC, and IGF1R, and thus triggers downstream signaling of MAPK, PI3K/AKT, STAT3, and NF-κB [4–6]. The cell surface receptors responsible for TFF3 function appear to be multiple and diverse with potential further TFF3 interacting proteins to be identified. Recently, CXCR4/7 [6], leucine-rich repeat receptor and nogo-interacting protein 2 (LINGO2) [7] and CD147 [8] have been implicated as potential receptors for TFF3.

Increasing evidence has indicated that TFF3 exerts crucial roles in the development and progression of various human cancers [4, 5, 9]. Indeed, TFF3 expression was reported to be elevated in colorectal [10–12], endometrial [13], gastric [14, 15], hepatocellular [16, 17], lung adeno- [18, 19], mammary [20–22], and prostate carcinomas [9, 23]. Increased TFF3 expression exerts pleiotropic effects that alter multiple biological processes involved in cancer development and progression. It has been demonstrated that TFF3 promotes cancer cell survival, proliferation, invasion, migration, angiogenesis, metastasis, and drug resistance [4, 18, 24–26]. In addition, increased expression of TFF3 is associated with poor prognosis of patients with various cancers, such as colorectal [27], gastric [14, 28, 29], hepatocellular [16], and mammary carcinomas [20, 30].

Whereas TFF3 has been suggested to exert pivotal functions in the development and progression of multiple cancers, the functions of TFF3 in PDAC have not been reported. Previous studies demonstrated significantly increased expression of TFF3 in human pancreatic carcinoma tissue [31, 32]. Similar results were also observed in the serum of patients with PDAC compared with benign controls [32]. Despite the above findings, the cellular functions, underlying molecular mechanisms, prognostic utility, and therapeutic potential of TFF3 in PDAC progression remain to be elucidated.

## Methods

### Cells and reagents

Cell lines utilized in this study, their source, and culture are listed in Supplementary Table 1. For the generation of stable cell lines, Capan-1 and Panc-1 cells were transfected with empty *pIRESneo3-vector* or *pIRESneo3-TFF3* plasmid, and SW1990 cells were transfected with the control siRNA plasmid or siRNA plasmid targeting *TFF3* as previously described [20]. CTNNB1 in SW1990 cells was depleted using siRNA (#19761 and #19762) from Addgene (Cambridge, MA, USA). FuGENE® HD Transfection Reagent used for plasmid transfection was purchased from Promega (Madison, WI, USA). AMPC was synthesized in the Department of Studies in Organic Chemistry, University of Mysore.

### Transcriptome analysis and tissue microarray

Transcriptome analysis of *TFF3* in human normal pancreatic tissues and PDAC was performed using the data from the publicly available database (Oncomine, https://www.oncomine.org/). The tissue microarray (HPanA120Su02) was obtained from Outdo Bio-tech Co., Ltd. (Shanghai, China). Consent for the use of the tissue samples and clinical data were obtained by Outdo Bio-tech Co., Ltd. (Shanghai, China). The clinicopathological information of all cases is publically available on the company website (http://www.superchip.com.cn/). Immunohistochemistry (IHC) staining and scoring were performed as previously described [33]. The corresponding antibodies used herein are listed in Supplementary Table 2. The staining results were assessed and confirmed by two independent researchers blinded to the clinical data.

### Quantitative PCR (qPCR)

qPCR was performed as previously described [33]. β-ACTIN mRNA was used as input control. The sequences of the oligonucleotide primers used herein are listed in Supplementary Table 3.

### RNA-seq

mRNA was isolated from cells using oligo(dT)-attached magnetic beads. RNA-seq was subsequently performed by BGI (Shenzhen, China) using the MGI2000 platform. The sequencing data were filtered with SOAPnuke (v1.5.2) followed by mapping to the reference genome using HISAT2 (v2.0.4). Differential expression analysis was performed using the DESeq2(v1.4.5) with *Q* value ≤0.05. Gene ontology (GO) and KEGG enrichment analysis were performed by Phyper based on a Hypergeometric test. Gene set enrichment analyses (GSEA) were performed using GSEA v3.0 software.

### Serum and cell-based assays

TFF3 concentrations were measured by Quantikine^®^ ELISA Human TFF3 Immunoassay kit (R&D Systems, USA) according to the manufacturer’s protocol. Western blot and immunofluorescence analysis were processed as previously described [33]. The corresponding antibodies used are listed in Supplementary Table 2. Cell function assays were performed as previously described [20, 33, 34].

### Xenografts

All animal experiments were approved by the Institutional Animal Care and Use Committee of the Laboratory Animal Centre of Peking University Shenzhen Graduate School (permit YW; the permit from Tsinghua Shenzhen International Graduate School is “Ethical Development no. 37 (year 2019)”. Six-week-old male BALB/c athymic nude mice were grouped by simple randomization using a random number table method. The principle of sample size followed the “The ARRIVE Guidelines” [35]. Mice were subcutaneously injected with Capan-1-ctrl/TFF3 (1 × 10^7^ cells) or SW1990-shctrl/shTFF3 (5 × 10^6^ cells). For drug treatment, the mice were subcutaneously injected with SW1990 (5 × 10^6^ cells). Six xenograft-bearing mice were each randomized into the vehicle, single-drug, double-drug, or triple-drug groups, and were treated with intraperitoneal injections of vehicle (1% DMSO/10% PEG400 in normal saline), 20 mg/kg AMPC, 10 mg/kg ICG-001 (Selleck Chemicals, USA), or 5 mg/kg gemcitabine (Eli Lily, USA). Xenograft volume was calculated as previously described [33]. Immunohistochemical analysis of xenograft histology sections was performed as previously described [30, 36].

### Statistical analysis

Two-tailed unpaired Student’s *t* test and ANOVA analysis were used to calculate the statistical significance of two or multiple treatment groups, respectively. As for overall survival analyses, Kaplan–Meier survival curves were used. The level of significance was set as *P < 0.05, **P < 0.01, and ***P < 0.001. Normally distributed data were presented as mean ± standard deviation.

## Results

### TFF3 expression is increased in PDAC

**Fig. 1:**
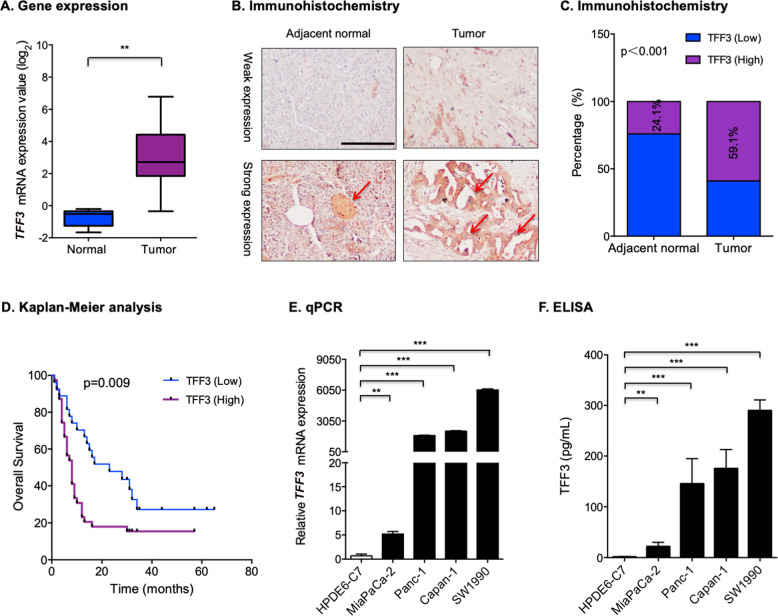
TFF3 expression was increased in PDAC. **A** Transcriptomic analysis of *TFF3* via a publicly available database (Oncomine). Data represent means ± SD. *P < 0.05, **P < 0.01, and ***P < 0.001. **B** IHC analysis for TFF3 protein expression in PDAC and normal tissues. Representative IHC images of TFF3 expression in adjacent normal and tumor tissues are shown. Scale bar, 20 μm. **C** The percentage of high versus low expression of TFF3 in the adjacent normal and tumor tissues. **D** Kaplan–Meier analysis of overall survival in PDAC stratified according to TFF3 low or high expression in PDAC tissues. **E** qPCR analysis for *TFF3* mRNA expression levels in human normal and PDAC cell lines. **F** ELISA analysis of extracellular TFF3 protein levels in human normal and PDAC cell lines.

*TFF3* mRNA expression in human normal pancreatic tissues and PDAC from the publicly available data set was first examined. It was observed that *TFF3* mRNA expression in PDAC was significantly higher than in normal pancreatic tissues (Fig. 1A). The expression levels of TFF3 protein in PDAC tissues and cell lines were next examined. By IHC analysis, it was observed that TFF3 protein exhibited significantly increased expression in PDAC compared with normal pancreatic tissues (Fig. 1B, C). A high expression level of TFF3 was observed in 59.1% (39/66) of cancer tissue, whereas in normal tissue, high expression was observed in 24.1% (13/54) of samples (Fig. 1C). Notably, in normal pancreatic tissue, TFF3 protein was mainly expressed in pancreatic islets (Fig. 1B), consistent with that reported in previous studies [37, 38]. However, the expression of TFF3 protein in cancer tissue was predominately located in PDAC cells (Fig. 1B). Furthermore, the correlation between TFF3 protein expression and the clinicopathologic characteristics of patients with PDAC was assessed. It was observed that TFF3 expression was not related to gender, age, or histological grade, but was significantly associated with tumor size (Supplementary Table 4). Importantly, survival analysis revealed that high TFF3 expression was positively correlated with worse overall survival of PDAC patients compared with those patients with low expression of TFF3 (Fig. 1D).

It was also demonstrated that there was significantly higher *TFF3* mRNA expression in human PDAC cell lines compared with that in the normal human pancreatic duct epithelial (HPDE6-C7) cell line (Fig. 1E). Considering that TFF3 is a secreted protein, the extracellular TFF3 protein levels were further determined. The results indicated that the PDAC cell lines exhibited higher concentrations of extracellular TFF3 protein compared with the HPDE6-C7 cell line (Fig. 1F). These findings provide further confirmatory evidence that TFF3 expression is increased in human PDAC.

### Forced expression of TFF3 promotes oncogenicity of PDAC cells

**Fig. 2:**
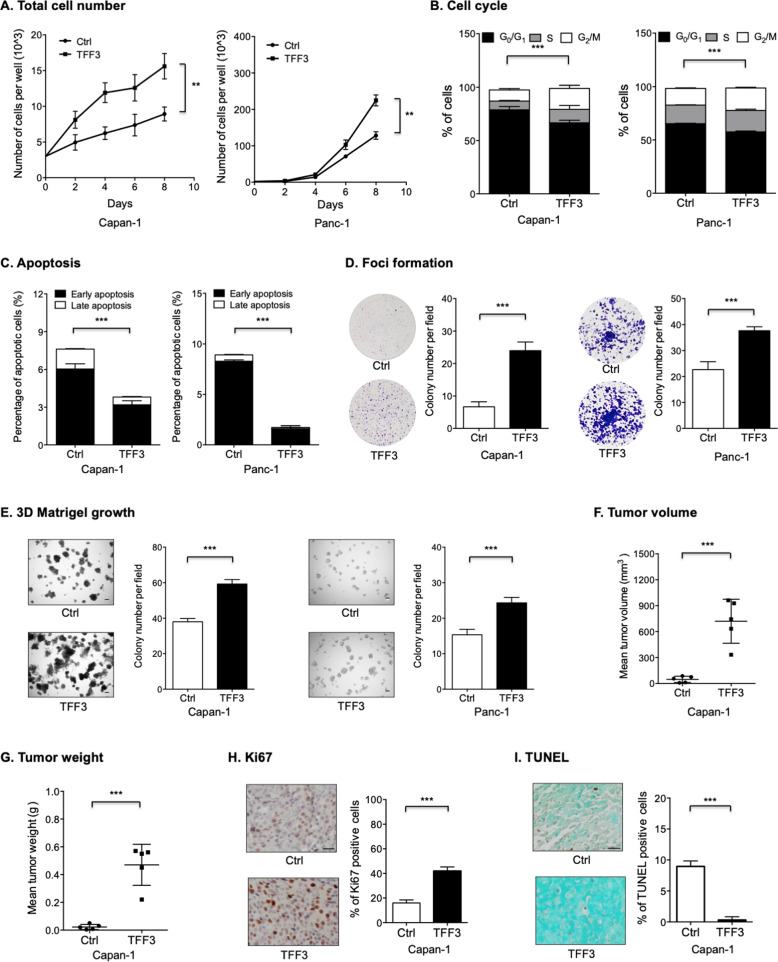
Forced expression of TFF3 promoted oncogenicity of PDAC cells. **A** Total cell number analysis was performed to determine the effect of forced expression of TFF3 on cell proliferation in Capan-1 and Panc-1 cells. **B** Cell-cycle progression change of Capan-1 and Panc-1 cells after forced expression of TFF3 was determined using PI staining followed by FACS analysis. The percentages of cells in each cell-cycle phase were plotted. **C** Apoptosis of Capan-1 and Panc-1 cells after forced expression of TFF3 was determined using AnnexinV/PI staining followed by FACS analysis. The percentages of cells in an early or late apoptotic phase were plotted. **D** Foci formation assay was performed to determine the effect of forced expression of TFF3 on foci formation by Capan-1 and Panc-1 cells. **E** 3D-Matrigel analysis was performed to determine the effect of forced expression of TFF3 on 3D growth of Capan-1 and Panc-1 cells. Scale bar, 50 μm. **F**–**G** The volume (**F**) and weight (**G**) of xenografts formed by Capan-1-TFF3 or the control cells at the termination of the experiment. **H** Representative micrographs and quantitative assay of IHC staining for Ki67 in the indicated xenografts. Scale bar, 20 μm. **I** Representative micrographs and quantitative assay of TUNEL staining in the indicated xenografts. Scale bar, 20 μm.

In order to determine the cellular function of TFF3 in PDAC progression, Capan-1 and Panc-1 cell lines with stable forced expression of TFF3 (designated as Capan-1-TFF3 and Panc-1-TFF3) were established. The enhanced expression of TFF3 was verified by qPCR and Western blot analysis (Fig. S1A, B, and SO1). Subsequently, the effect of forced expression of TFF3 on PDAC cell proliferation was examined by total cell number and BrdU incorporation assays. Forced expression of TFF3 enhanced the proliferative capability of Capan-1 cells compared with the control cells (Figs. 2A and S1C). Similar results were observed in Panc-1 cells (Figs. 2A and S1C). It was also observed that forced expression of TFF3 in Capan-1 and Panc-1 cells markedly promoted cell-cycle progression and suppressed apoptosis (Figs. 2B, C, S1D, E). Moreover, forced expression of TFF3 increased foci formation of Capan-1 and Panc-1 cells (Fig. 2D). Three-dimensional (3D) Matrigel assays were used to examine the effect of forced expression of TFF3 on the 3D growth of PDAC cells. The colonies formed by Capan-1–TFF3 and Panc-1–TFF3 cells were significantly more numerous and larger compared with the respective control cells, suggesting that forced expression of TFF3 promoted 3D growth of PDAC cells (Fig. 2E). In addition, Capan-1–TFF3 cells also exhibited a significant increase in migration and invasion compared with the control cells (Fig. S1F, G).

Next, it was determined whether forced expression of TFF3 accelerated PDAC growth in vivo using a mouse xenograft model. Forced expression of TFF3 significantly increased the xenograft volume and weight at the termination of the experiment (Fig. 2F,G). The retention of increased expression of TFF3 in xenografts derived from Capan-1-TFF3 cells was demonstrated by IHC analysis (Fig. S1H). IHC analysis further revealed that forced expression of TFF3 in PDAC cells markedly increased the proportion of Ki67-positive cells (Fig. 2H). In addition, forced expression of TFF3 in PDAC cells also significantly reduced apoptosis (Fig. 2I). Hence, forced expression of TFF3 facilitates PDAC progression in vitro and in vivo.

### Depletion of TFF3 suppresses oncogenicity of PDAC cells

**Fig. 3:**
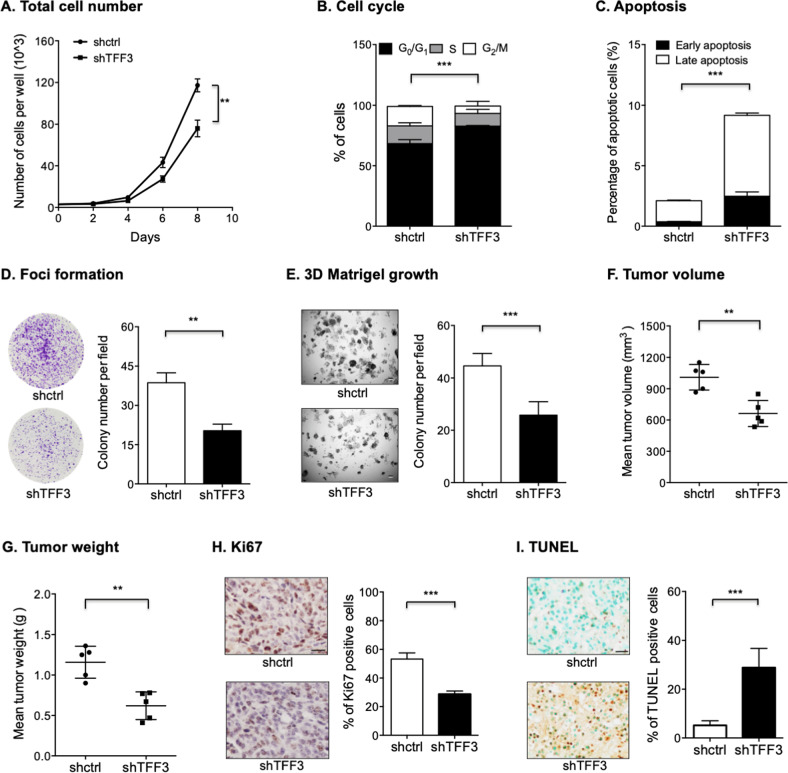
Depletion of TFF3 suppressed oncogenicity of PDAC cells. **A** Total cell number analysis was performed to determine the effect of depletion of TFF3 on cell proliferation in SW1990 cells. **B** Cell-cycle progression of SW1990 cells after depletion of TFF3 was determined using PI staining followed by FACS analysis. The percentages of cells in each cell-cycle phase were plotted. **C** Apoptosis of SW1990 cells after depletion of TFF3 was determined using AnnexinV/PI staining followed by FACS analysis. The percentages of cells in an early or late apoptotic phase were plotted. **D** Foci formation assay was performed to determine the effect of depletion of TFF3 on foci formation of SW1990 cells. **E** 3D-Matrigel analysis was performed to determine the effect of depletion of TFF3 on 3D growth of SW1990 cells. Scale bar, 50 μm. **F**–**G** The volume (**F**) and weight (**G**) of xenografts formed by SW1990-shTFF3 and the control cells at the termination of the experiment. **H** Representative micrographs and quantitative assay of IHC staining results for Ki67 in the indicated xenografts. Scale bar, 20 μm. **I** Representative microscopic pictures and quantitative assay of TUNEL staining results in the indicated xenografts. Scale bar, 20 μm.

*TFF3* was depleted in SW1990 cells via a siRNA-based approach (designated as SW1990-shTFF3). The depletion efficiency of TFF3 was determined by qPCR and western blot analysis (Fig. S2A, B, and SO2). It was observed that depletion of TFF3 significantly inhibited the proliferation of SW1990 cells (Figs. 3A and S2C). Depletion of TFF3 in SW1990 cells also significantly suppressed cell-cycle progression and promoted apoptosis (Figs. 3B, C, S2D, E). Furthermore, depletion of TFF3 impaired foci formation and 3D growth of SW1990 cells (Fig. 3D, E) as well as decreased migration and invasion (Fig. S2F, G).

As shown in Fig. 3F, G, depletion of TFF3 in SW1990 cells significantly reduced xenograft volume and weight at the termination of the experiment. The retention of TFF3 depletion in xenografts was confirmed using IHC analysis (Fig. S2H). IHC analysis further revealed that depletion of TFF3 in SW1990 cells resulted in reduced proliferation and increased apoptosis of cancer cells (Fig. 3H, I). Hence, depletion of TFF3 suppresses PDAC progression in vitro and in vivo.

### AMPC suppresses the oncogenicity of PDAC cells

TFF3 has been reported to form disulfide-linked dimers with itself or other proteins via the seventh cysteine (Cys57) residue [10, 39]. The monomeric and dimeric forms of TFF3 exhibit differences in cellular functions [39]. Notably, the homodimeric form of TFF3 was reported to be essential for the pro-proliferative and anti-apoptotic functions of TFF3 [10]. A small-molecule inhibitor of TFF3 (designated as AMPC) that monomerizes homodimeric TFF3 was previously generated [10, 18].

**Fig. 4:**
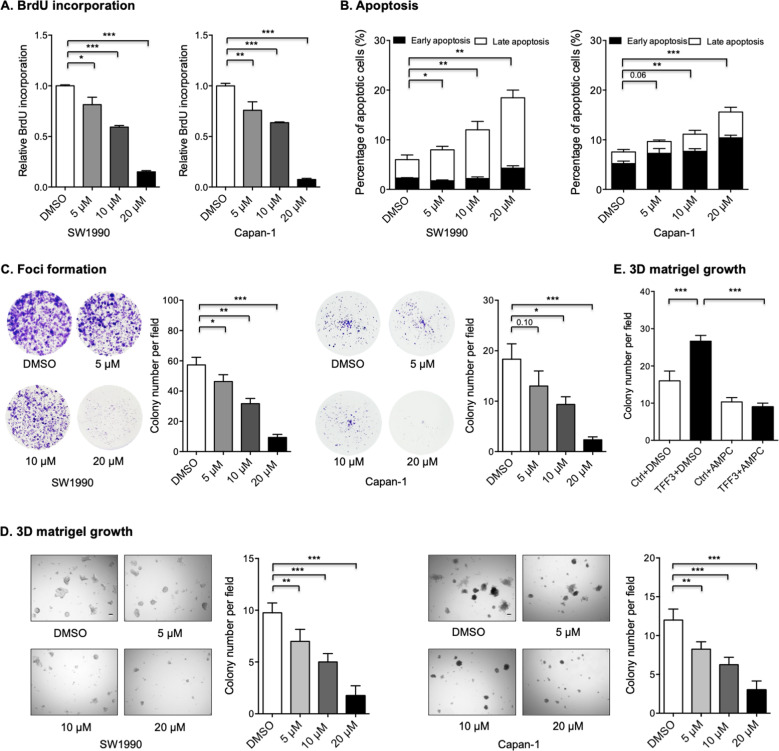
AMPC suppressed oncogenicity of PDAC cells. **A** BrdU assay showed that AMPC treatment for 48 hours inhibited the proliferative capability of SW1990 and Capan-1 cells in a dose-dependent manner. **B** Apoptosis after AMPC treatment for 48 hours was determined using AnnexinV/PI staining followed by FACS analysis. The percentages of cells in the early or late apoptotic phase are plotted. **C** Foci formation assay showed that AMPC treatment for attenuated foci formation of SW1990 and Capan-1 cells in a dose-dependent manner. **D** 3D-matrigel growth analysis showed that AMPC treatment attenuated 3D growth of SW1990 and Capan-1 cells in a dose-dependent manner; **E** 3D-matrigel growth analysis showed that AMPC treatment abrogated the increased 3D growth produced by forced expression of TFF3 in Capan-1 cells.

The IC_50_ value of AMPC in multiple PDAC cell lines was determined using total cell number assays. It was observed that the IC_50_ value of AMPC in SW1990 cells with the highest endogenous TFF3 expression was significantly lower than those in other cell lines (Figs. 1E and S3A). AMPC treatment significantly impaired the proliferative capability of SW1990 cells in a dose-dependent manner (Fig. 4A). Similar results were observed in Capan-1 cells (Fig. 4A). It was also observed that AMPC treatment markedly increased apoptosis of SW1990 and Capan-1 cells (Figs. 4B and S3B). In addition, AMPC treatment significantly impaired foci formation and 3D growth of PDAC cells (Fig. 4C, D), similar to that previously observed after depletion of TFF3 (Fig. 3D, E). Notably, AMPC treatment attenuated the increased 3D growth capacity resulting from forced expression of TFF3 in Capan-1 cells (Fig. 4E). Hence, pharmacological inhibition of TFF3 suppresses the oncogenicity of PDAC cells.

### TFF3 induces WNT ligand expression in PDAC

**Fig. 5:**
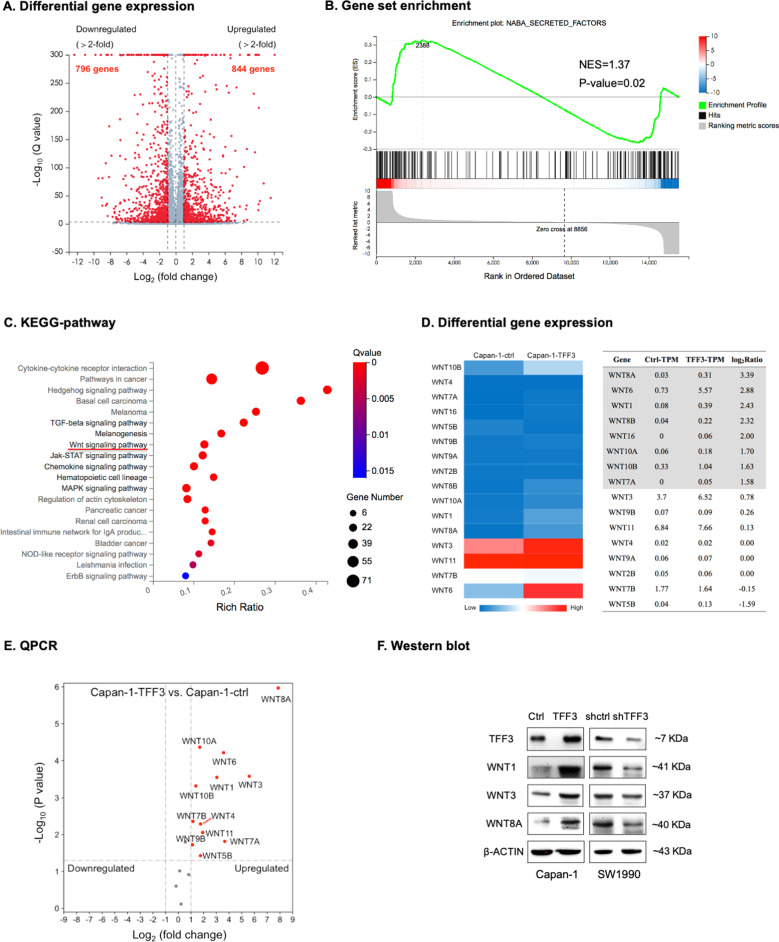
TFF3 induced WNT ligand expression in PDAC. **A** Volcano plot shows the differentially up- or downregulated genes between Capan-1-TFF3 and the control cells. Genes deemed statistically significant are highlighted in red. **B** Relevant gene set enrichment plots from gene set enrichment analysis of secreted factors (NES, normalized enrichment score). **C** KEGG-pathway analysis was performed to identify the molecular pathways with highly altered activities. **D** Heatmap for differential expression of WNT ligand genes between Capan-1-TFF3 and the control cells. **E** Volcano plot of the gene expression profile of WNT ligands in Capan-1-TFF3 and the control cells based on qPCR. Mean expression relative to β-actin was plotted according to the log_2_ fold change (*X* axis) and log_10_ *P* value (*Y* axis). **F** Western blot analysis was performed to determine the protein expression level of WNT ligands in Capan-1-TFF3, SW1990-shTFF3, and the respective control cells. β-actin was used as input control.

To further explore the potential signaling mechanisms responsible for the oncogenic functions of TFF3 in PDAC progression, RNA-sequencing (RNA-seq) was performed to characterize the transcriptome of Capan-1-TFF3 cells in comparison to the control cells. Gene expression analysis identified 1640 differentially expressed genes (fold change > 2), including 796 downregulated genes and 844 upregulated genes in Capan-1-TFF3 cells (Fig. 5A). GO analysis further indicated that many modulated genes were associated with the extracellular space (Fig. S4A). Consistently, gene set enrichment analysis further revealed that the genes with differential expression showed significant enrichment for “secreted factors” and “core matrisome” (Figs. 5B and S4B), both of which are closely associated with the extracellular space. Furthermore, KEGG-pathway analysis identified highly altered expression in several pathways; the most altered pathways included Hedgehog, TGF-β, and WNT signaling pathways (Fig. 5C). As the WNT pathway has been shown to possess critical functions in PDAC progression [40], WNT-associated alterations in Capan-1-TFF3 cells were investigated. It was observed that multiple WNT ligands, such as *WNT1/6/7A/8A*/*8B*/*10A/10B&16*, exhibited significantly increased expression in Capan-1-TFF3 cells compared with those in the control cells (Fig. 5D), suggesting that TFF3 enhanced WNT ligand expression.

qPCR was, therefore, performed to verify WNT ligand mRNA expression changes after forced expression of *TFF3* in Capan-1 cells. As shown in Fig. 5E, forced expression of *TFF3* in Capan-1 cells significantly increased the expression of multiple WNT ligands, including *WNT1*/*3*/*4B*/*6/7A/7B/8A/9B/10A/10B&11*. In addition, western blot analysis showed that forced expression of *TFF3* in Capan-1 cells also enhanced the expression of WNT1/3&8A proteins (as exemplars), whereas depletion of *TFF3* in SW1990 cells reduced their protein expression (Fig. 5F and SO3). These results were consistent with that observed in RNA-seq, verifying that TFF3 promotes WNT ligand expression.

### TFF3 activates WNT signaling by inducing WNT ligand expression in PDAC

**Fig. 6:**
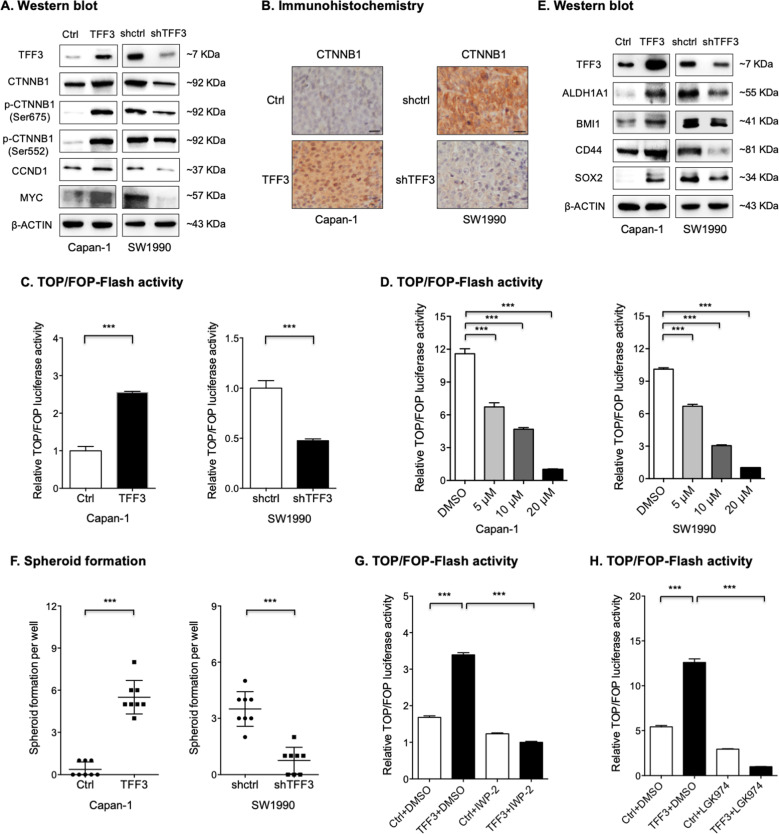
TFF3 activated WNT signaling by inducing WNT ligand expression in PDAC. **A** Western blot analysis was performed to determine the expression of WNT-related proteins in Capan-1-TFF3, SW1990-shTFF3, and the corresponding control cells. β-actin was used as input control. **B** Representative micrographs of IHC staining for CTNNB1 in the indicated xenografts. Scale bar, 20 μm. **C** Relative TOP/FOP luciferase activity analysis was performed to determine the change of CTNNB1/TCF transcription activity in Capan-1-TFF3, SW1990-shTFF3, and the respective control cells. **D** Relative TOP/FOP luciferase activity analysis was performed to determine the change of CTNNB1/TCF transcription activity in Capan-1 and SW1990 cells after AMPC treatment. **E** Western blot analysis was performed to determine the protein expression of stemness-associated genes in Capan-1-TFF3, SW1990-shTFF3, and the corresponding control cells. β-actin was used as input control. **F** Spheroid formation assay for Capan-1-TFF3, SW1990-shTFF3, and the respective control cells. **G**, **H** TOP/FOP luciferase activity analysis was performed to determine the change of CTNNB1/TCF transcription activity in Capan-1-TFF3 and the control cells after the suppression of WNT ligand secretion with 10 μM IWP-2 (**G**) or LGK-974 (**H**) treatment for 48 hours.

It was further investigated whether TFF3 activates WNT signaling in PDAC. As shown in Fig. 6A, S5A, and SO4, it was observed that forced expression of *TFF3* in Capan-1 cells increased the protein expression of CTNNB1 (β-CATENIN), a key component of the canonical WNT pathway, while depletion of *TFF3* in SW1990 cells reduced its expression. This finding was further supported by IHC staining of CTNNB1 protein in xenografts generated in the xenograft models described above (Fig. 6B). A positive correlation between the protein expression levels of TFF3 and CTNNB1 in PDAC cell lines (Fig. S5B and SO5) was also observed. In addition, forced expression of TFF3 in Capan-1 cells also increased the levels of phosphorylated forms of CTNNB1 (Ser675 and Ser552) presumed to be the active forms of CTNNB1 (Fig. 6A). Consistent results were obtained in SW1990 cells after depletion of TFF3 or after AMPC treatment (Figs. 6A, S5C, and SO6). As shown by luciferase reporter activity (TOP/FOP Flash) assays, forced expression of TFF3 in Capan-1 cells also activated CTNNB1/TCF transcriptional activity, while depletion of TFF3 in SW1990 cells suppressed this transcriptional activity (Fig. 6C). This was corroborated by the decreased CTNNB1/TCF transcriptional activity observed in Capan-1 and SW1990 cells after AMPC treatment (Fig. 6D). As shown in Fig. 6A, forced expression of TFF3 significantly increased the protein expression of WNT target genes CCND1 and MYC, whereas depletion of TFF3 reduced their expression. Consistent results were obtained in SW1990 cells after AMPC treatment (Fig. S5C and SO6). Hence, TFF3 activates the WNT pathway signaling in PDAC cells.

It has been reported that the WNT pathway promotes cancer stem cell (CSC)-like behavior in multiple cancers [40–42], and hence it was next determined whether TFF3 regulates CSC-like behavior in PDAC. As shown in Supplementary Fig. 5D, forced expression of TFF3 in Capan-1 cells significantly increased the mRNA expression of several common stemness-associated genes, such as *BMI1*, *CD9*, *CD44,* and *SOX2* [43, 44]. Conversely, depletion of TFF3 in SW1990 cells attenuated the expression of these genes at the mRNA level (Fig. S5D). The expression changes of stemness-associated genes were further confirmed at the protein level (Fig. 6E and SO7). In addition, forced expression of TFF3 significantly promoted the spheroid formation and the tumor-initiating capacity of Capan-1 cells, whereas depletion of TFF3 in SW1990 cells significantly impaired these properties (Figs. 6F and S5E). Hence, TFF3 promotes CSC-like behavior in PDAC cells.

It was further determined whether TFF3 activates WNT signaling by inducing WNT ligand expression in PDAC. IWP-2 and LGK-974 are two small-molecule inhibitors, both of which block WNT ligand secretion by inhibiting PORCN enzymatic activity [45]. As shown in Fig. 6G, IWP-2 treatment attenuated the increased CTNNB1/TCF transcriptional activity of Capan-1 cells observed with forced expression of TFF3. Similar results were observed after LGK-974 treatment (Fig. 6H). Hence, TFF3 activates the WNT signaling pathway by inducing WNT ligand expression.

### TFF3 promotes oncogenic functions of PDAC cells in a WNT-dependent manner

**Fig. 7:**
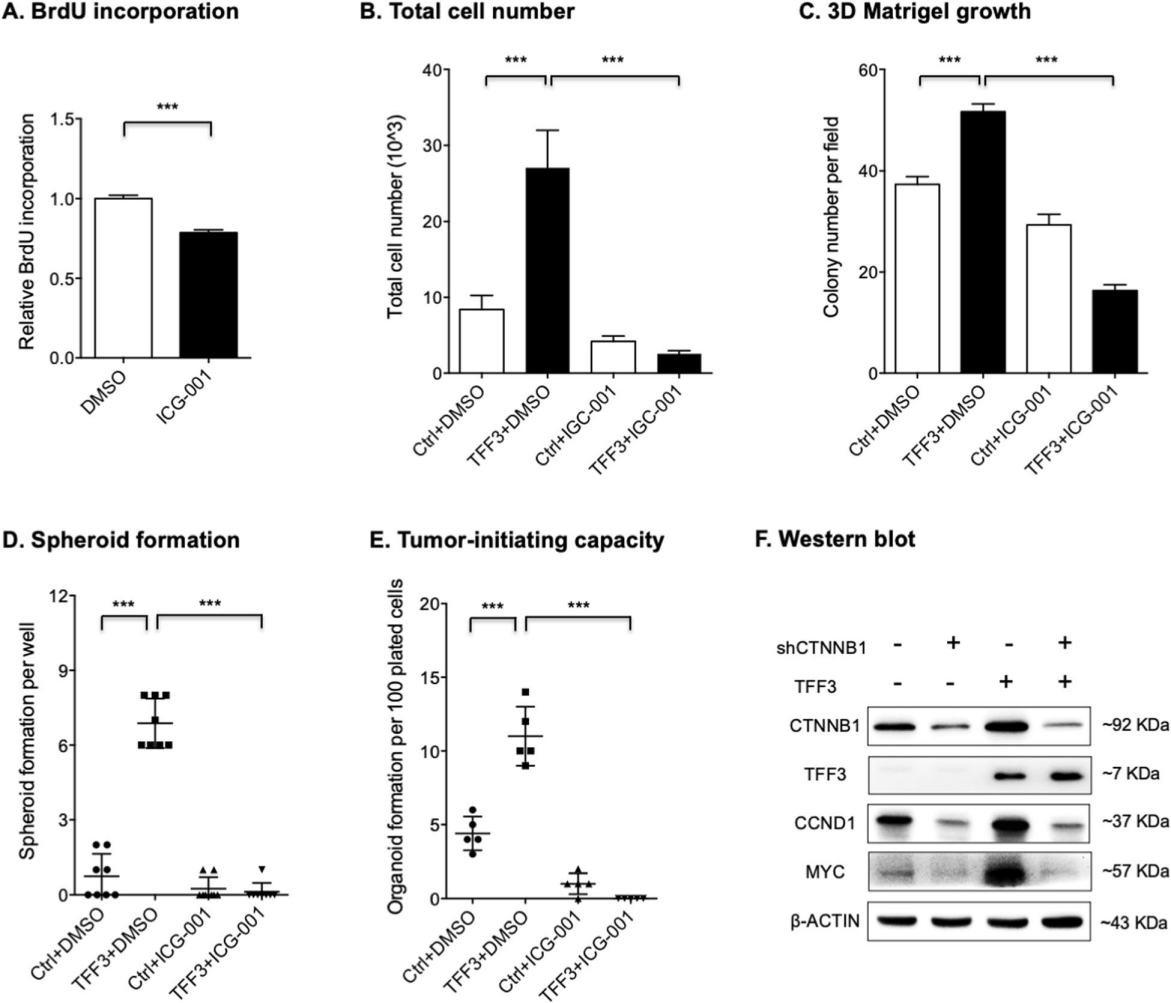
TFF3 promoted oncogenic functions of PDAC cells in a WNT-dependent manner. **A** BrdU analysis for SW1990 cells treated with 2 μM ICG-001 for 48 hours. **B**, **C** Total cell number counting (**B**) and 3D-Matrigel (**C**) analysis showed that forced expression of TFF3 in Capan-1 cells significantly promoted cell proliferation and 3D growth, respectively, and 2 μM ICG-001 treatment for 48 hours abrogated these processes. **D**, **E** Spheroid (**D**) and organoid (**E**) formation assay showed that forced expression of TFF3 in Capan-1 cells significantly promoted colony formation and tumor-initiating capabilities respectively, and 2 μM ICG-001 treatment for 48 hours reversed these processes. **F** Western blot analysis showed that depletion of CTNNB1 prevented the upregulation of WNT-associated gene expression produced by forced expression of TFF3 in Capan-1 cells. β-actin was used as input control.

Previous studies have reported that WNT signaling promotes PDAC progression [40, 46]. Consistently, depletion of CTNNB1 via RNA interference resulted in significantly decreased proliferation of SW1990 cells (Fig. S6A, B, C, and SO8). Similar effects were also observed in SW1990 cells treated with ICG-001 (Fig. 7A), a common small-molecule inhibitor of WNT pathway that functions by specifically inhibiting CTNNB1/TCF transcriptional activity. As observed in Fig. 7B, treatment with ICG-001 abrogated the increased proliferation of Capan-1 cells with forced expression of TFF3. Consistently, ICG-001 treatment also decreased the enhanced 3D growth of Capan-1-TFF3 cells compared with that in the control cells (Fig. 7C). In addition, it was observed that ICG-001 treatment abrogated the enhanced spheroid formation and tumor-initiating capacities of Capan-1 cells with forced expression of TFF3 (Fig. 7D, E). Western blot analysis further indicated that depletion of CTNNB1 prevented the increased WNT-associated gene expression produced by forced expression of TFF3 in Capan-1 cells (Fig. 7F and SO9). Hence, TFF3 promotes the oncogenic function of PDAC cells in a WNT-dependent manner.

### Combination treatment of AMPC and WNT inhibitor suppresses xenograft growth and potentiates the efficacy of gemcitabine

**Fig. 8:**
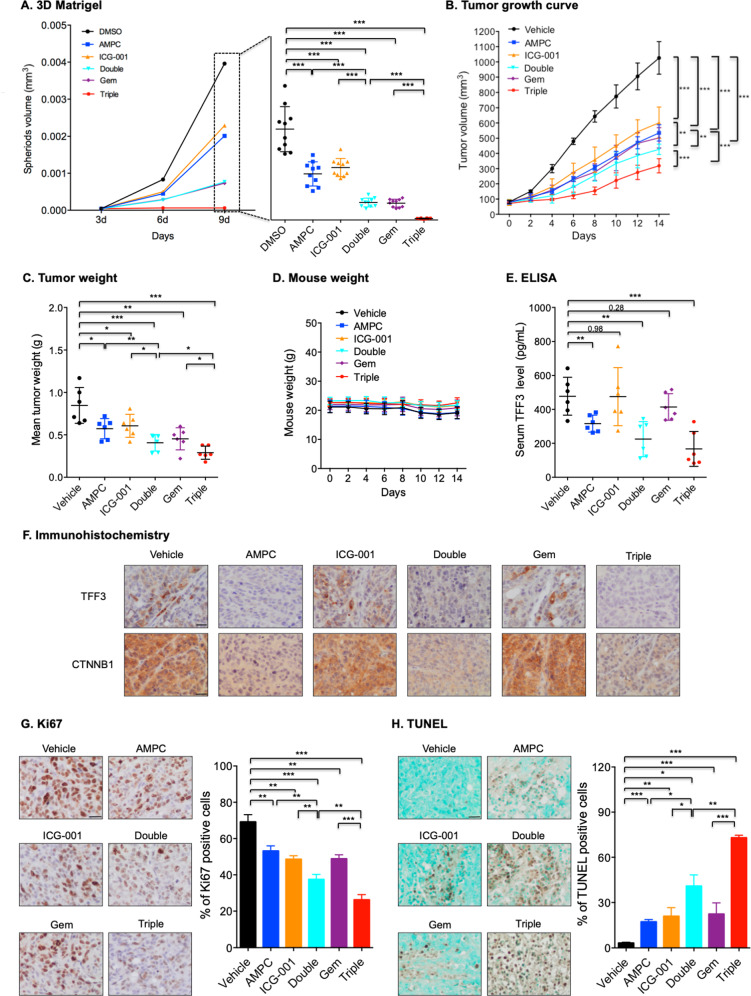
Combination treatment of AMPC and WNT inhibitor suppressed xenograft growth and potentiated the efficacy of gemcitabine. **A** 3D-Matrigel growth analysis for SW1990 cells after AMPC, ICG-001, AMPC + ICG-001 (Double), gemcitabine (Gem) or AMPC + ICG-001 + Gem (Triple) treatment. The graph shows the spheroid volume in each group. Data represent means ± SD from at least three independent experiments. *P < 0.05, **P < 0.01, and ***P < 0.001. **B** Growth curve of SW1990-derived xenografts after treatment with vehicle, AMPC, ICG-001, Double, Gem, or Triple. *n* = 6 mice per group. Data are represented as means ± SEM. *P < 0.05, **P < 0.01, and ***P < 0.001. **C** Weights of the isolated SW1990-derived xenografts at the termination of the experiments. **D** Body weights of the mice during the treatment period. *n* = 6 mice per group. Data are represented as mean ± SD. *P < 0.05, **P < 0.01, and ***P < 0.001. **E** ELISA analysis for serum levels of human TFF3 derived from xenografts. **F** Representative micrographs of IHC staining for TFF3 and CTNNB1 in the indicated xenografts. Scale bar, 20 μm. **G** Representative micrographs and quantitative assay of IHC staining results for Ki67 in the indicated xenografts. Scale bar, 20 μm. Data are represented as means ± SD. *P < 0.05, **P < 0.01, and ***P < 0.001. **H** Representative micrographs and quantitative assay of TUNEL staining results in the indicated xenografts. Scale bar, 20 μm. Data are represented as means ± SD. *P < 0.05, **P < 0.01, and ***P < 0.001.

Combination therapy improves therapeutic efficacy compared with single-drug treatment by enhancing cytotoxicity and reducing the development of drug resistance in cancer cells [47, 48]. The therapeutic efficacy of AMPC in combination with ICG-001 was, therefore, first determined in a 3D-Matrigel growth model. The established colonies were treated with AMPC, ICG-001, or a combination of AMPC and ICG-001. On the 9th day, a significant difference in the colony volume was observed in the AMPC-treated group compared with the vehicle group (Fig. 8A). Moreover, combination treatment of AMPC and ICG-001 led to smaller colony volumes compared with colonies treated with only AMPC or ICG-001 alone (Fig. 8A).

Gemcitabine is a current standard of therapy for PDAC. However, drug resistance and side effects have seriously limited its therapeutic efficacy [49, 50]. Therefore, it was investigated whether a combination treatment of AMPC and ICG-001 would enhance the inhibitory effect of gemcitabine. It was observed that the triple combination of AMPC, ICG-001, and gemcitabine significantly reduced the colony volume compared with colonies co-treated with AMPC and ICG-001 or treated with a single drug (Fig. 8A), indicating that combination treatment of AMPC and ICG-001 enhanced the efficacy of gemcitabine in PDAC.

It was next determined whether the enhanced efficacy of combination treatment with AMPC and ICG-001 would also be observed in the SW1990 xenograft model. Xenograft-bearing mice were randomized into the vehicle, single-drug, double-drug, or triple-drug groups, and were treated with intraperitoneal injections of vehicle, AMPC, ICG-001, gemcitabine, or combination treatment. After 2 weeks of treatment, AMPC treatment significantly inhibited xenograft growth compared with the vehicle group (Fig. 8B and Supplementary Fig. 7A), similar to that previously observed after depletion of TFF3 (Fig. 3F, G). Furthermore, combination treatment with AMPC and ICG-001 exhibited significantly stronger effects on xenograft growth inhibition than the vehicle or single-drug group (Fig. 8B). The triple combination of AMPC, ICG-001, and gemcitabine resulted in the highest inhibition of xenograft growth compared with any other group (Fig. 8B). These results were also confirmed by the measurement of xenograft weight at the termination of the experiments (Fig. 8C). The drug treatments were well tolerated, as observed by lack of significant change of body weight and the weights of representative major organs, including liver, kidney, and lung (Fig. 8D and Supplementary Fig. 7A).

AMPC treatment reduces cellular and secreted TFF3 levels [18, 24]. To determine the efficacy of AMPC, the serum levels of human TFF3 derived from xenograft animals were therefore measured. As shown in Fig. 8E, all groups treated with AMPC exhibited lower levels of TFF3 compared with groups without AMPC treatment, including the single ICG-001 or gemcitabine group with reduced xenograft volume. Similar results were obtained by IHC analysis for TFF3 in xenograft tissue samples (Fig. 8F). AMPC treatment also significantly reduced CTNNB1 expression in xenograft tissues compared with non-treated controls (Fig. 8F). Combination treatment with AMPC and ICG-001 resulted in significantly reduced xenograft cell proliferation and increased apoptosis compared with the vehicle and single-drug groups (Figs. 8G, H). The triple combination of AMPC, ICG-001, and gemcitabine led to the lowest percentage of Ki67-positive cells and the highest percentage of TUNEL-positivity compared with other groups (Fig. 8G,H). Hence, combination treatment of AMPC and ICG-001 suppresses xenograft growth and potentiates the therapeutic efficacy of gemcitabine in vivo.

## Discussion

There are still no FDA-approved drugs that effectively target the most commonly mutated genes (*KRAS*, *CDKN2A*, *TP53,* and *SMAD4*) in PDAC, although Sotorasib (a KRAS-G12C inhibitor) was recently approved for advanced non-small cell lung cancer [1, 51]. Hence, characterization of novel potent and non-mutated oncogenic drivers that contribute to PDAC progression is necessary to potentially improve targeted therapeutic outcomes. Consistent with recent studies [31, 32], TFF3 exhibited an elevated expression in PDAC compared with that in normal tissues. The data herein further revealed that TFF3 expression in PDAC, regardless of underlying genetic mutations, was positively correlated with worse overall survival, implying that TFF3 may function as an independent prognostic indicator for PDAC.

AMPC was used herein to determine the potential efficacy of pharmacological targeting TFF3 in PDAC. It was observed that AMPC significantly impaired PDAC cell number increases in vitro and in vivo, consistent with previous observations [10, 18, 24]. Hence, precision targeting of TFF3 may be a potential therapeutic strategy for PDAC. It should be noted that compared with ER^+^ mammary carcinoma cells, AMPC appeared less efficacious in the PDAC cell lines used herein. However, considering the limited options that are available for PDAC treatment, this study may expand therapeutic opportunities for PDAC.

Recently, several studies reported that TFF3 promoted CSC-like properties in the colorectal, hepatocellular, lung, and mammary carcinoma [16, 18, 20]. However, the mechanism by which TFF3 promotes CSC-like properties remained to be elucidated. It was observed herein that TFF3 activated the WNT signaling pathway by enhancing WNT ligand expression. Hence, at least in PDAC, TFF3 might promote CSC-like behavior by activating the WNT pathway. There are still no FDA-approved drugs that target the WNT pathway for cancer therapy. Inhibition of the WNT pathway produces considerable side effects as the WNT pathway is critical for the maintenance of normal stem cells [52]. Hence, combination therapy with a low-dose WNT inhibitor and other therapeutic agents may prove to be more clinically useful [53]. In the present study, combination treatment with AMPC and WNT inhibitor exhibited significantly stronger effects on xenograft growth inhibition than the vehicle or single-drug groups. In addition, it was observed that the combination treatments were well tolerated.

In conclusion, the oncogenic function of TFF3 in PDAC progression has been systematically elucidated herein. Considering the paucity of knowledge of the specific determinants promoting PDAC development/progression, this study provides novel insights on TFF3 regulation of PDAC cell function and provides a rationale for considering the use of TFF3 inhibitors in the therapy of PDAC.

## Supplementary information

Supplementary Figures and Tables

Supplementary Original Western Blots

Reproducibility checklist

## Data Availability

The data sets used in this study are available from the corresponding author on reasonable request. Requests for materials should be addressed to Vijay Pandey or Peter E. Lobie.
