# Supplementary material for: Trefoil factor 3 promotes pancreatic carcinoma progression via WNT pathway activation mediated by enhanced WNT ligand expression
Source: Cell Death Dis. 2022 Mar 25;13(3):265. doi: 10.1038/s41419-022-04700-4 (PMC8948291; doi:10.1038/s41419-022-04700-4)
Supplement: Supplementary file 1 — Supplementary Figures and Tables [file 41419_2022_4700_MOESM1_ESM.pdf]

Trefoil factor 3 promotes pancreatic carcinoma progression via WNT pathway activation mediated by enhanced WNT ligand expression

Feifei Cheng, Xuejuan Wang, Yi-Shiou Chiou, Chuyu He, Hui Guo, Yan Qin Tan, Basappa, Tao Zhu, Vijay Pandey and Peter E. Lobie

Supplementary Figures and Tables

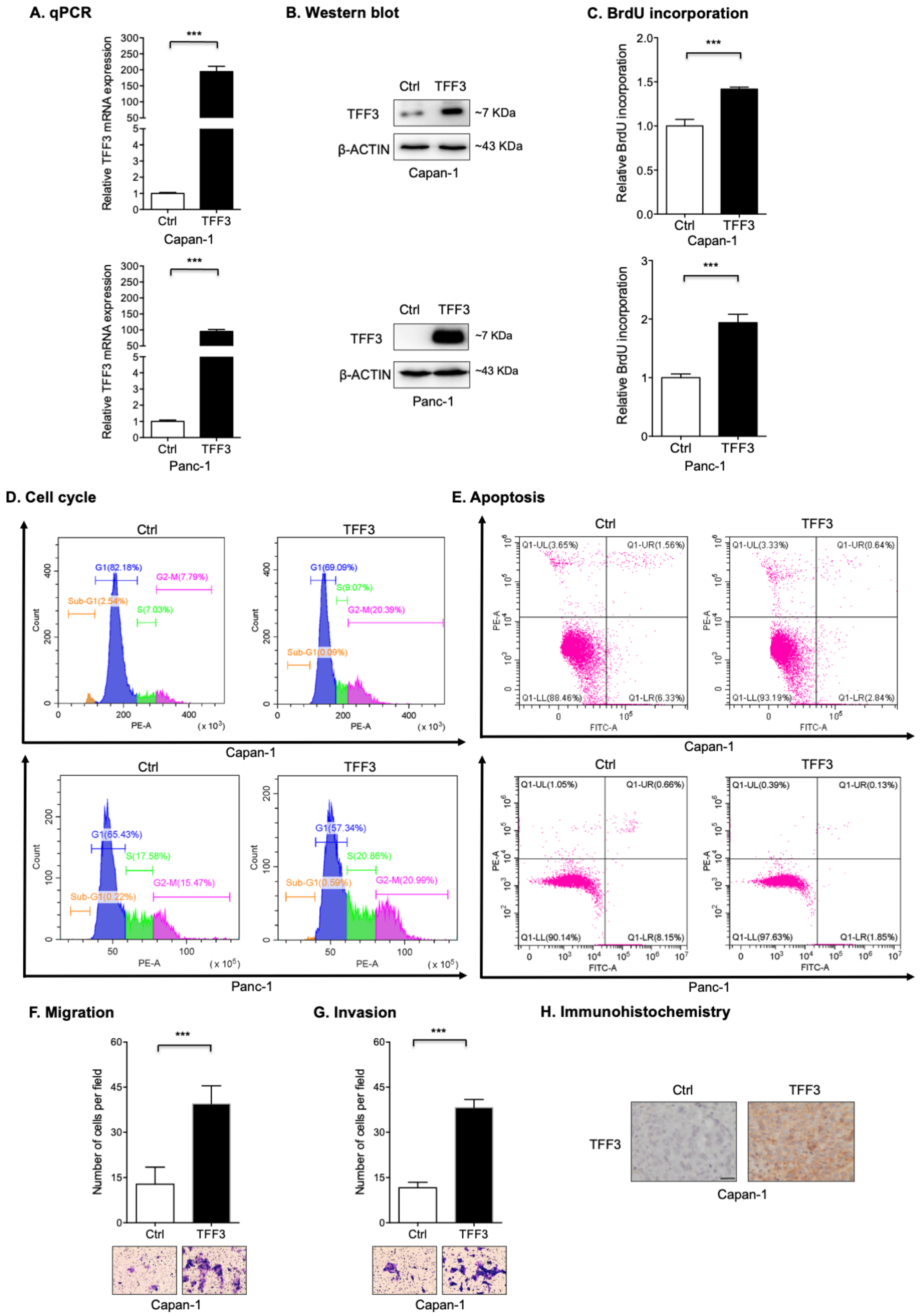

Fig. S1 Forced expression of TFF3 promoted oncogenicity of PDAC cells. (A and B) qPCR (A) and Western blot (B) analysis were performed to confirm the forced expression of TFF3 in Capan-1-TFF3 and Panc-1-TFF3 cells. β-ACTIN was used as input control. (C) BrdU assay was performed to determine the effect of forced

expression of TFF3 on S-phase entry in Capan-1 and Panc-1 cells. (D) Representative FACS images of cell-cycle progression change after forced expression of TFF3 in Capan-1 and Panc-1 cells. (E) Representative FACS images of apoptosis after forced expression of TFF3 in Capan-1 and Panc-1 cells. (F and G) Transwell analysis was performed to determine the effect of forced expression of TFF3 on cell migration (F) and invasion (G) in Capan-1 cells. (H) Representative micrographs of IHC staining for TFF3 in the indicated xenografts. Scale bar, 20 μm.

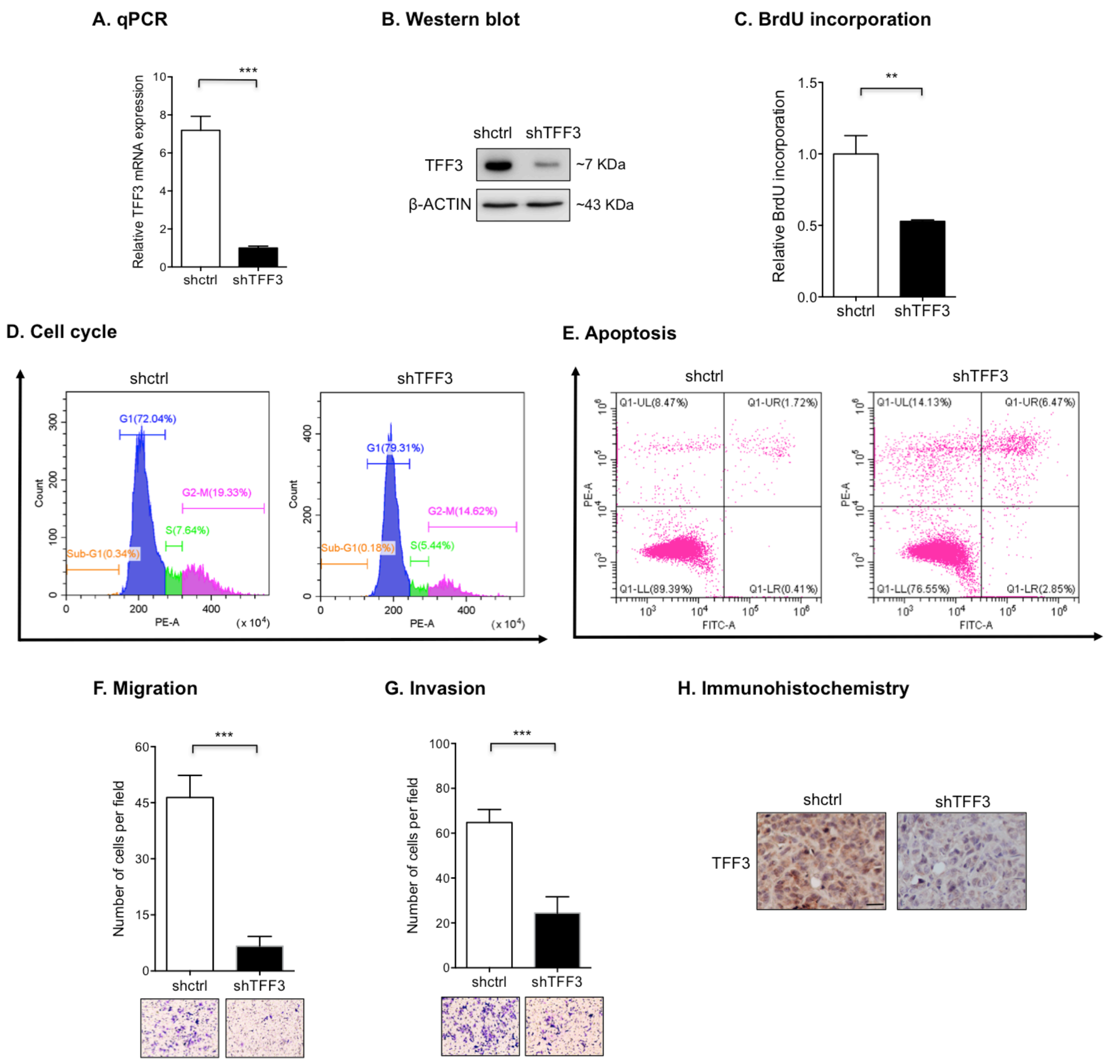

Fig. S2 Depletion of TFF3 suppressed oncogenicity of PDAC cells. (A and B) qPCR (A) and Western blot (B) analysis were performed to confirm the depletion efficiency of TFF3 in SW1990-shTFF3 cells. β-ACTIN was used as input control. (C) BrdU assay was performed to determine the effect of depletion of TFF3 on S-phase entry in SW1990 cells. (D) Representative FACS images of cell-cycle progression change after depletion of TFF3 in SW1990 cells. (E) Representative FACS images of apoptosis after depletion of TFF3 in SW1990 cells. (F and G) Transwell analysis was performed to determine the effect of depletion of TFF3 on cell migration (F) and invasion (G) in SW1990 cells. (H) Representative micrographs of IHC staining for TFF3 in the indicated xenografts. Scale bar, 20 µm.

A. IC_{50} Value

Cell lines	$IC_{50}\pm SD$ (μM)
MiaPaCa-2	13.04±1.08
Capan-1	10.16±1.28
Panc-1	8.41±1.26
SW1990	5.38±1.13

B. Apoptosis

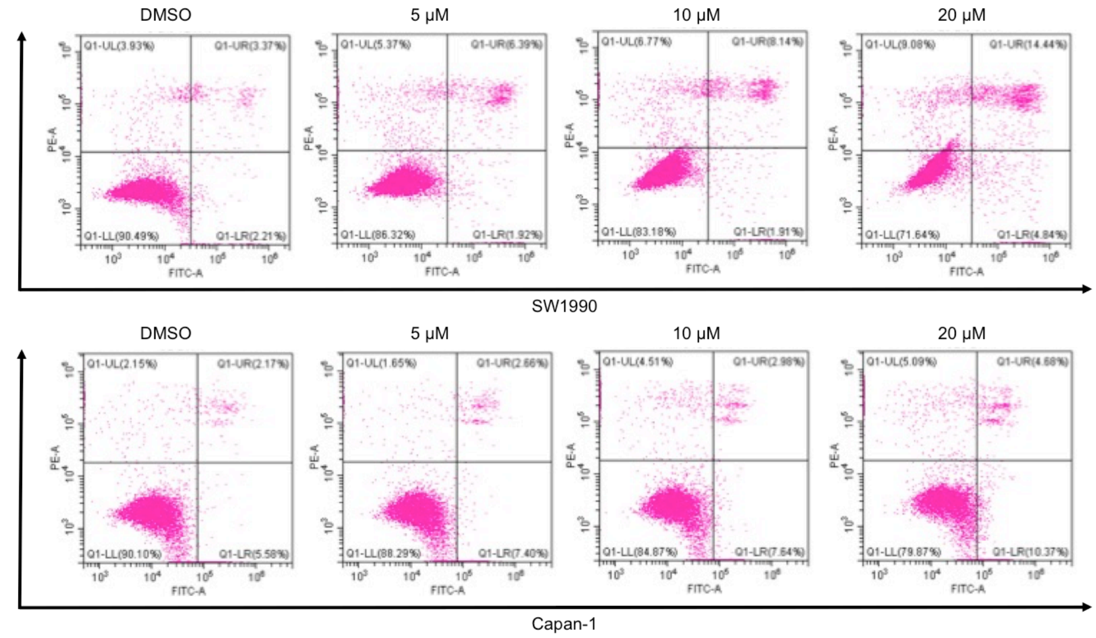

Fig. S3 AMPC suppressed oncogenicity of PDAC cells. (A) IC_{50} values of AMPC for multiple PDAC cell lines. (B) Representative images of apoptosis after AMPC treatment in SW1990 and Capan-1 cells.

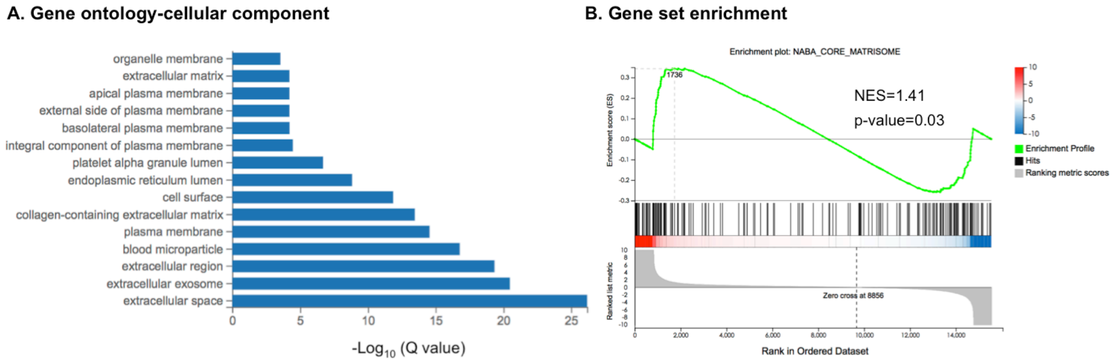

Fig. S4 TFF3 induced WNT ligand expression in PDAC. (A) Gene ontology analysis shows the cellular components most altered between the compared groups. (B) Relevant gene set enrichment plots from gene set enrichment analysis of core matrisome (NES, normalized enrichment score).

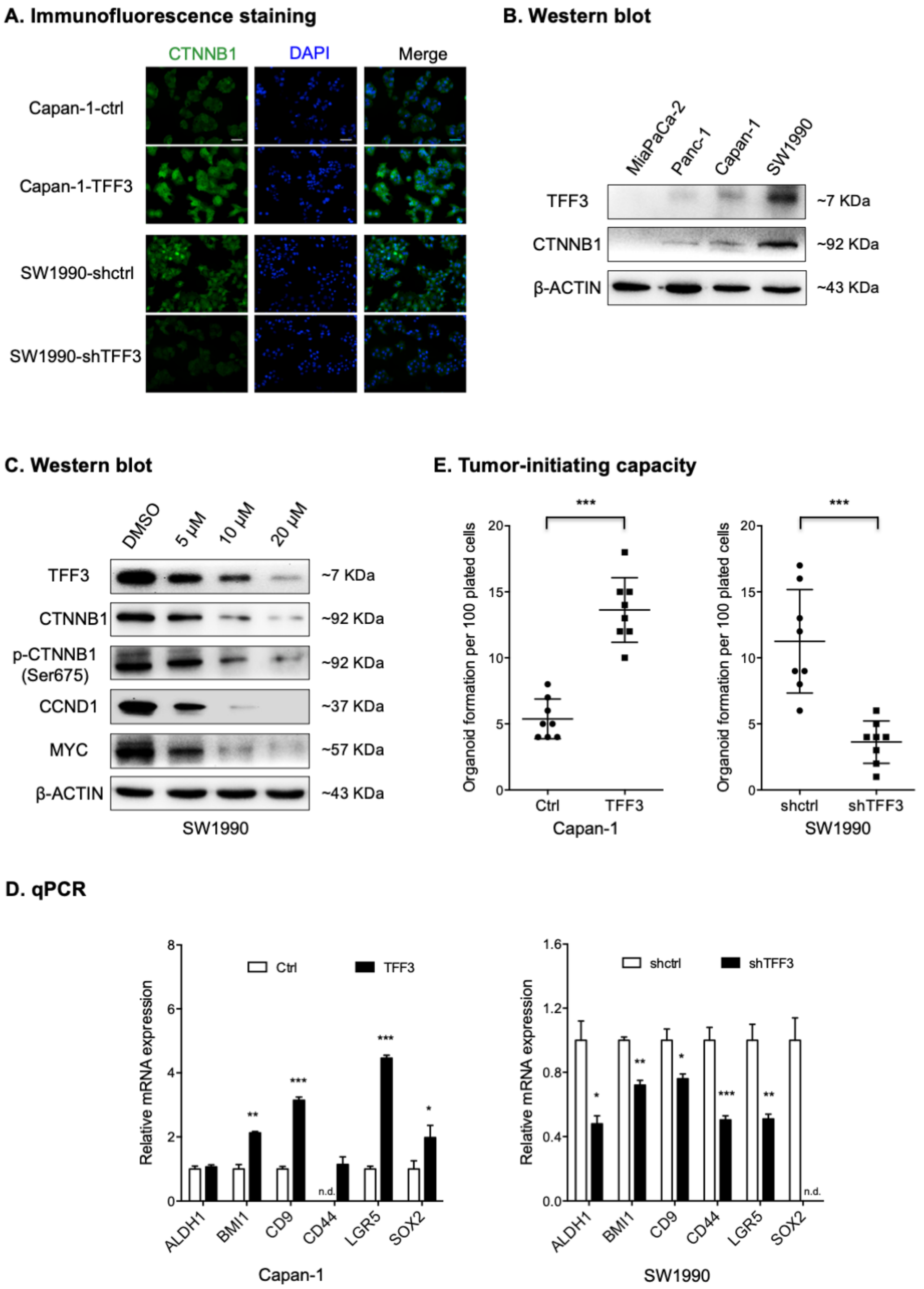

Fig. S5 TFF3 activated WNT pathway by inducing WNT ligand expression in PDAC. (A) Immunofluorescence analysis was performed to determine the expression change of CTNNB1 (β-CATENIN) in Capan-1-TFF3, SW1990-shTFF3 and the corresponding

control cells. (B) Western blot analysis was performed to determine the protein expression levels of TFF3 and CTNNB1 (β-CATENIN) in MiaPaCa-2, Panc-1, Capan-1 and SW1990 cells. β-ACTIN was used as input control. (C) Western blot analysis was performed to determine the protein levels of TFF3 and WNT-associated genes in SW1990 cells after AMPC treatment. β-ACTIN was used as input control. (D) qPCR analysis was performed to determine the relative expression of stemness-associated genes at the mRNA level in Capan-1-TFF3, SW1990-shTFF3 and the corresponding control cells. (E) Organoid formation assay was performed to determine tumor-initiating capacity in Capan-1-TFF3, SW1990-shTFF3 and the respective control cells.

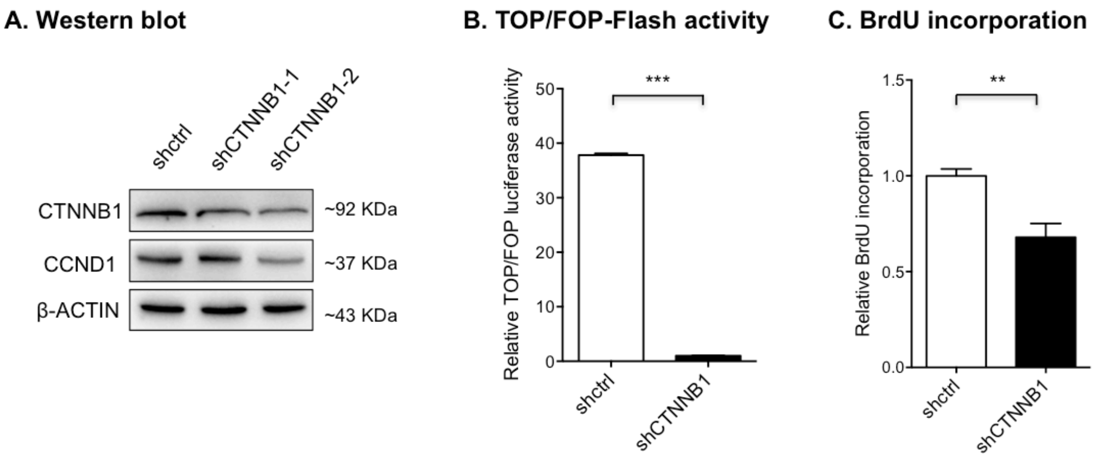

Fig. S6 TFF3 promoted oncogenic function of PDAC cells in a WNT-dependent manner. (A) Western blot analysis for CTNNB1 and CCND1 protein expression in SW1990 cells transfected with control shRNA, CTNNB1 shRNA1 or 2. β-ACTIN was used as input control. (B) TOP/FOP luciferase activity analysis for SW1990 cells with depletion of CTNNB1. (C) BrdU incorporation analysis for SW1990 cells with depletion of CTNNB1.

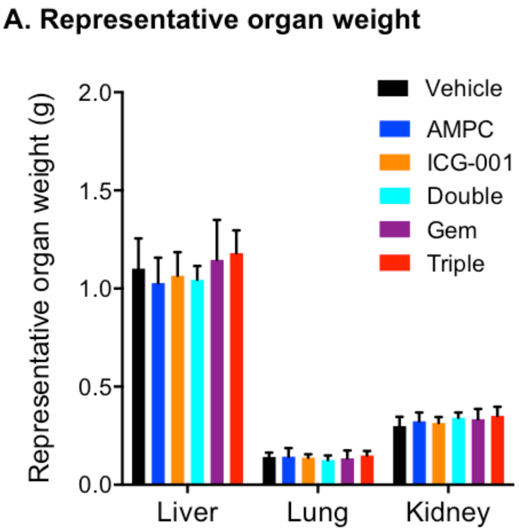

Fig. S7 Representative organ weights of the mice from the indicated groups at the termination of the experiment. n = 6 mice per group.

Supplementary Table 1：Cell lines used in this study

Cell line	Source	Culture	Mycoplasma test
Capan-1	ATCC	RPMI 1640 + 10% FBS	Negative
Mia Paca-2	FuHeng Biology Tech Co., Ltd.	DMEM + 10% FBS	
Panc-1	Procell Life Science & Technology Co., Ltd.		
SW1990			
HPDE6-C7	BNBio Tech Co., Ltd.		

Supplementary Table 2： Antibodies used in this study

Antibody	Assays	Company	Catalog	Dilution
TFF3	WB/IHC	Abcam	ab108599	1:1000/1:100
Ki67	IHC	Abcam	ab16667	1:500
CTNNB1	WB/IF/IHC	Cell Signaling Technology	#8480	1:1000/1:100/1:100
p-CTNNB1 (Ser675)	WB	Cell Signaling Technology	#4176	1:1000
p-CTNNB1 (Ser552)	WB	Cell Signaling Technology	#5651	1:1000
CCND1	WB	Santa Cruz	sc-8376	1:1000
MYC	WB	Abcam	ab32072	1:1000
ALDH1A1	WB	Cell Signaling Technology	#36671	1:1000
BMI1	WB	Cell Signaling Technology	#6964	1:1000
CD44	WB	Abcam	ab157107	1:1000
LGR5	WB	Omnimabs	OM283436	1:1000
SOX2	WB	Abcam	ab97959	1:1000
ZO-1	WB	Abcam	ab96587	1:1000
WNT1	WB	Abcam	ab15251	1:1000
WNT3	WB	Abcam	ab32249	1:1000
WNT8A	WB	Novus Biologicals	23050002	1:5000
β-ACTIN	WB	Santa Cruz	sc-47778	1:1000

Notes: WB—Western blot; IF—Immunofluorescence; IHC—Immunohistochemistry

Supplementary Table 3: Oligonucleotide primers used for qPCR

Gene	Forward primer	Reverse primer
TFF3	5′- CTTGCTGTCCTCCAGCTCT-3′	5′- CCGGTTGTTGCACTCCTT-3′
ALDH1	5′- GCACGCCAGACTTACCTGTC-3′	5′- CCTCCTCAGTTGCAGGATTAAAG-3′
BMI1	5′- GCTGCCAATGGCTCTAATGAA-3′	5′- TGCTGGGCATCGTAAGTATCTT-3′
CD9	5′- TCACCATGCCGGTCAAAGGA-3′	5′- GCCGGCTCCGATCAGAATAT-3′
CD44	5′- CCCATCCCAGACGAAGACAG-3′	5′- ACCATGAAAACCAATCCCAGG-3′
LGR5	5′- CTCCCAGGTCTGGTGTGTTG-3′	5′- GAGGTCTAGGTAGGAGGTGAAG-3′
SOX2	5′- GCCGAGTGGAAACTTTTGTCG-3′	5′- GGCAGCGTGTACTTATCCTTCT-3′
WNT1	5′- GGTGGGGTATTGTGAACGTAG-3′	5′- CGTATCAGACGCCGCTGTTT-3′
WNT2B	5′- GTTACCCAGACATCATGCGTT-3′	5′- GGGTGGTACAGTTCCAGCG-3′
WNT3	5′- CTCGCTGGCTACCCAATTTG-3′	5′- AGGCTGTCATCTATGGTGGTG-3′
WNT4	5′- CTCCACACTCGACTCCTTGC-3′	5′- CCGAAGAGATGGCGTACACG-3′
WNT5B	5′- GTGCAGAGACCCGAGATGTTT-3′	5′- TTGGCTCCCTCCCCTATGTAG-3′
WNT6	5′- GGTGCGAGAGTGCCAGTTC-3′	5′- CGTCTCCCGAATGTCCTGTT-3′
WNT7A	5′- CTGTGGCTGCGACAAAGAGAA-3′	5′- GCCGTGGCACTTACATTCC-3′
WNT7B	5′- GAAGCAGGGCTACTACAACCA-3′	5′-CGGCCTCATTGTTATGCAGGT-3′
WNT8A	5′- GAACTGCCCTGAAAATGCTCT-3′	5′- TCGAAGTCACCCATGCTACAG-3′
WNT9A	5′- AGCAGCAAGTTCGTCAAGGAA-3′	5′- CCTTCACACCCACGAGGTTG-3′
WNT9B	5′- TGTGCGGTGACAACCTCAAG-3′	5′- ACAGGAGCCTGATACGCCAT-3′
WNT10A	5′- GGTCAGCACCCAATGACATTC-3′	5′- TGGATGGCGATCTGGATGC-3′
WNT10B	5′- CATCCAGGCACGAATGCGA-3′	5′- CGGTTGTGGGTATCAATGAAGA-3′
WNT16	5′- AGTATGGCATGTGGTTCAGCA-3′	5′- GCGGCAGTCTACTGACATCAA-3′
β-ACTIN	5′-AGAAAATCTGGCACCACACC-3′	5′-AGAGGCGTACAGGGATAGCA-3′

Supplementary Table 4: Correlation between TFF3 protein expression and the clinicopathologic characteristics of patients with PDAC.

Variables	Total		TFF3 (High)		TFF3 (Low)		P-value
	N	%	N	%	N	%	
Gender							1.00
Male	37	56.1	22	56.4	15	55.6	
Female	29	43.9	17	43.6	12	44.4	
Age							0.613
＜65	28	42.4	18	46.2	10	37.0	
⩾65	38	57.6	21	53.8	17	63.0	
Tumor size							**0.016**
＜5 cm	43	65.2	21	53.8	22	81.5	
⩾5 cm	21	31.8	17	43.6	4	14.8	
NA	2	3.0	1	2.6	1	3.7	
Histological grade							0.688
I	5	7.6	3	6.4	2	10.5	
II	53	80.3	39	83.0	14	73.7	
III	8	12.1	5	10.6	3	15.8	
TNM stage							0.141
I-II	58	87.8	33	84.6	25	92.6	
III-IV	4	6.1	4	10.3	0	0	
NA	4	6.1	2	5.1	2	7.4	
