# Supplementary material for: Trefoil factor 3 promotes pancreatic carcinoma progression via WNT pathway activation mediated by enhanced WNT ligand expression
Source: Cell Death Dis. 2022 Mar 25;13(3):265. doi: 10.1038/s41419-022-04700-4 (PMC8948291; doi:10.1038/s41419-022-04700-4)
Supplement: Supplementary file 2 — Supplementary Original Western Blots [file 41419_2022_4700_MOESM2_ESM.pdf]

Trefoil factor 3 promotes pancreatic carcinoma progression via WNT pathway activation mediated by enhanced WNT ligand expression

Feifei Cheng, Xuejuan Wang, Yi-Shiou Chiou, Chuyu He, Hui Guo, Yan Qin Tan, Basappa, Tao Zhu, Vijay Pandey and Peter E. Lobie

Supplementary Original Western Blots

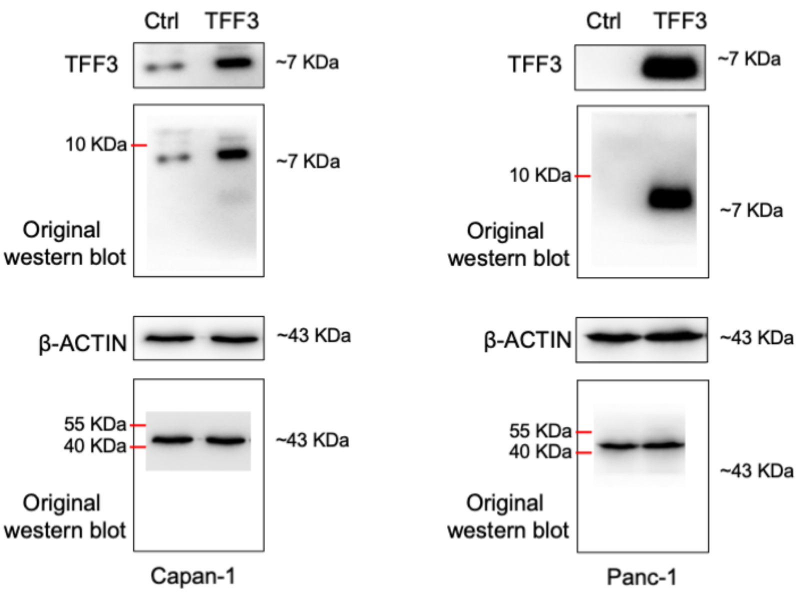

Fig. SO1 Original western blots for the western blot result in Fig. S1B

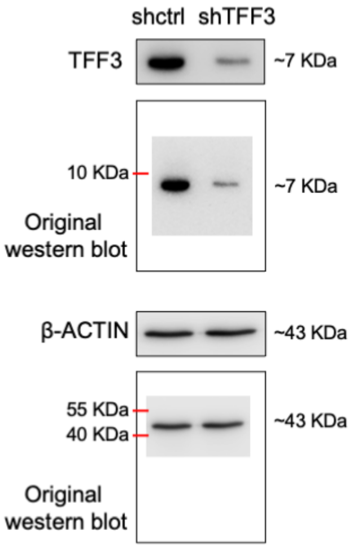

Fig. SO2 Original western blots for the western blot result in Fig. S2B

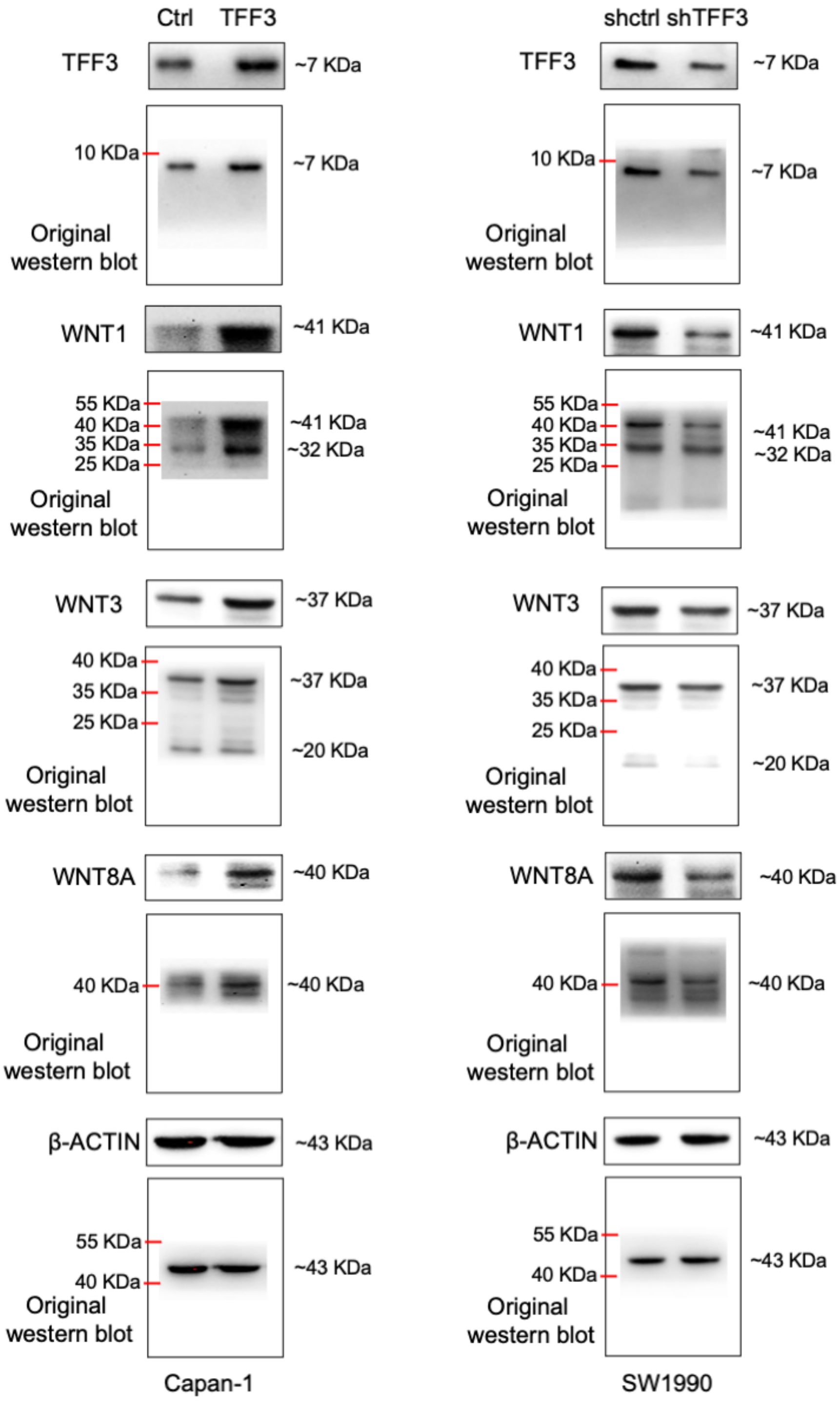

Fig. SO3 Original western blots for the western blot result in Fig. 5F

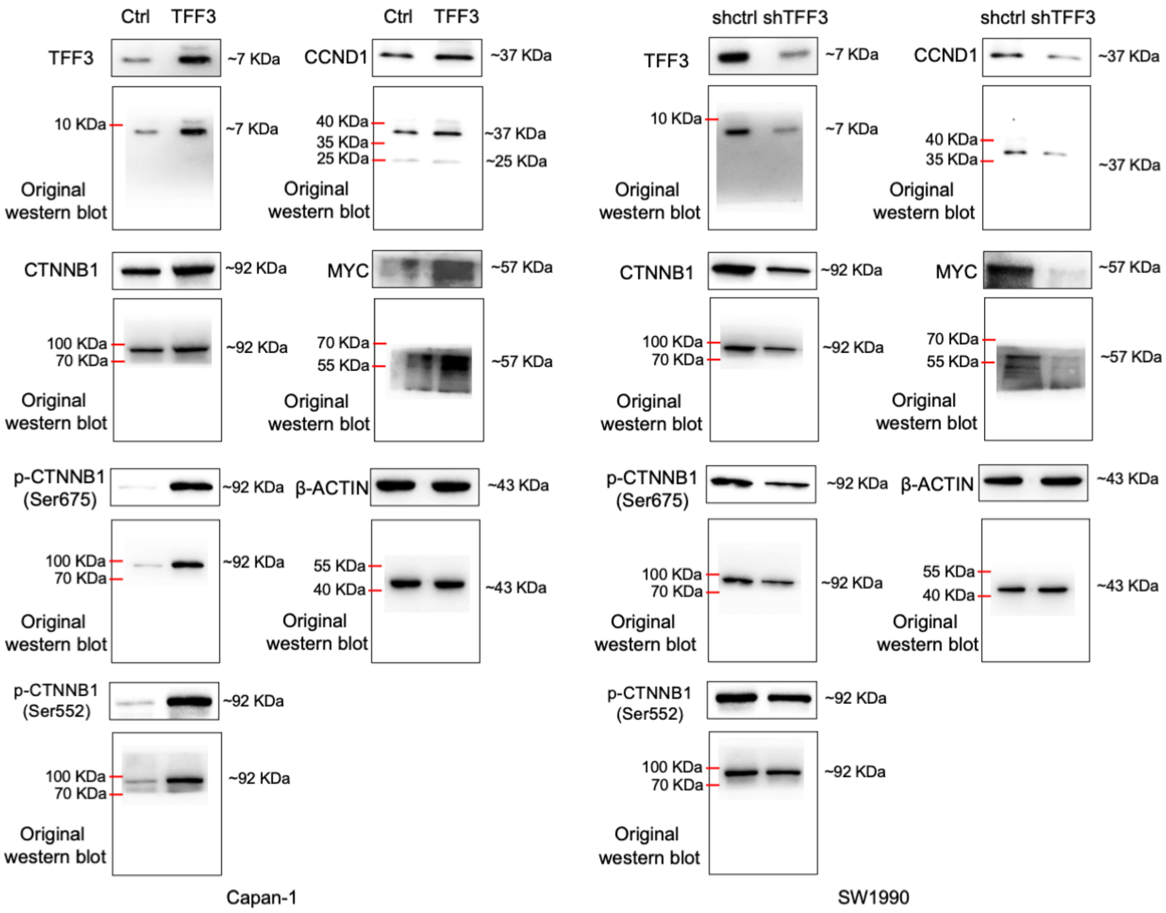

Fig. SO4 Original western blots for the western blot result in Fig. 6A

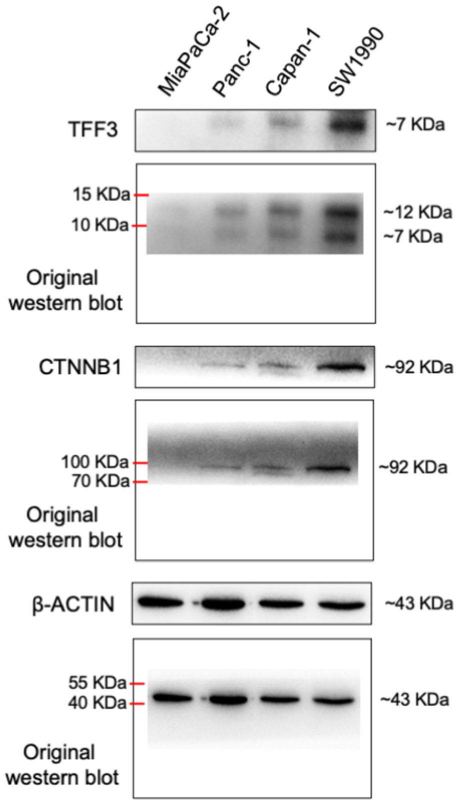

Fig. SO5 Original western blots for the western blot result in Fig. S5B

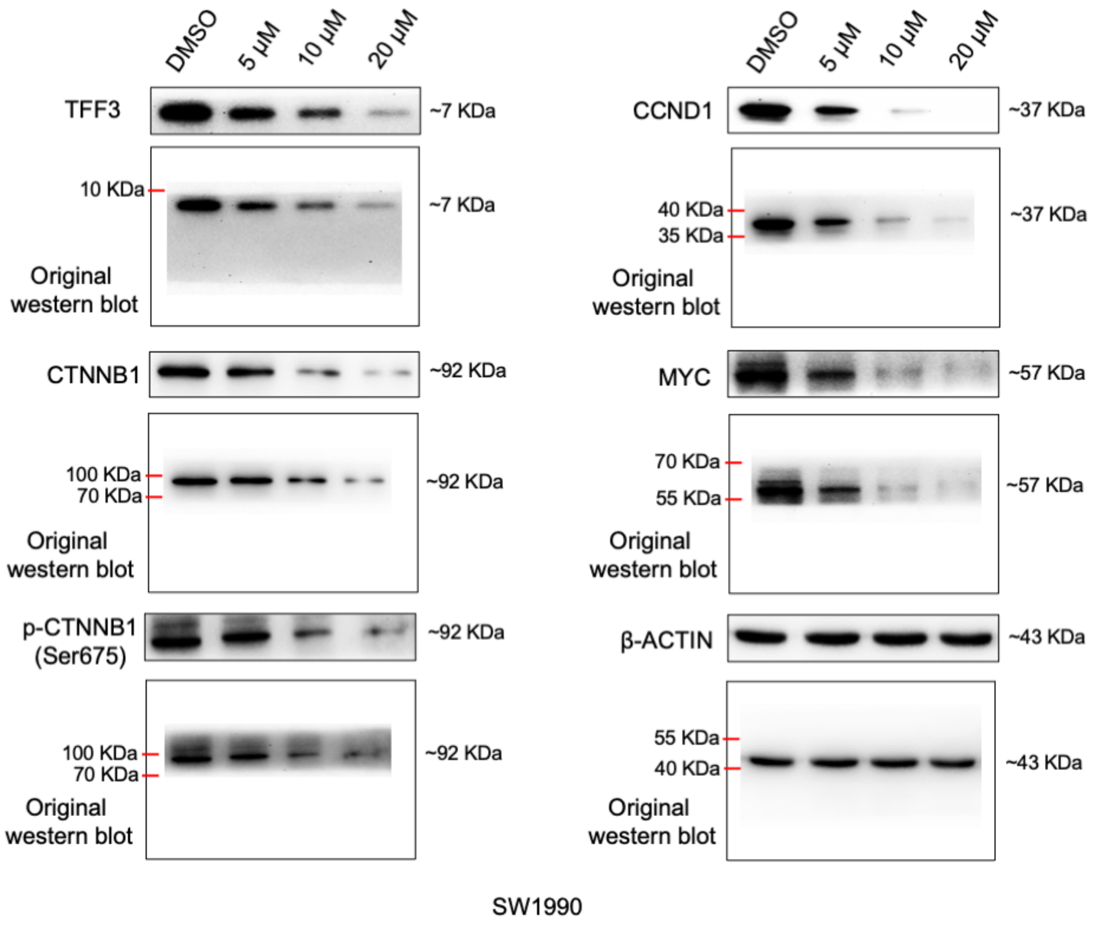

Fig. SO6 Original western blots for the western blot result in Fig. S5C

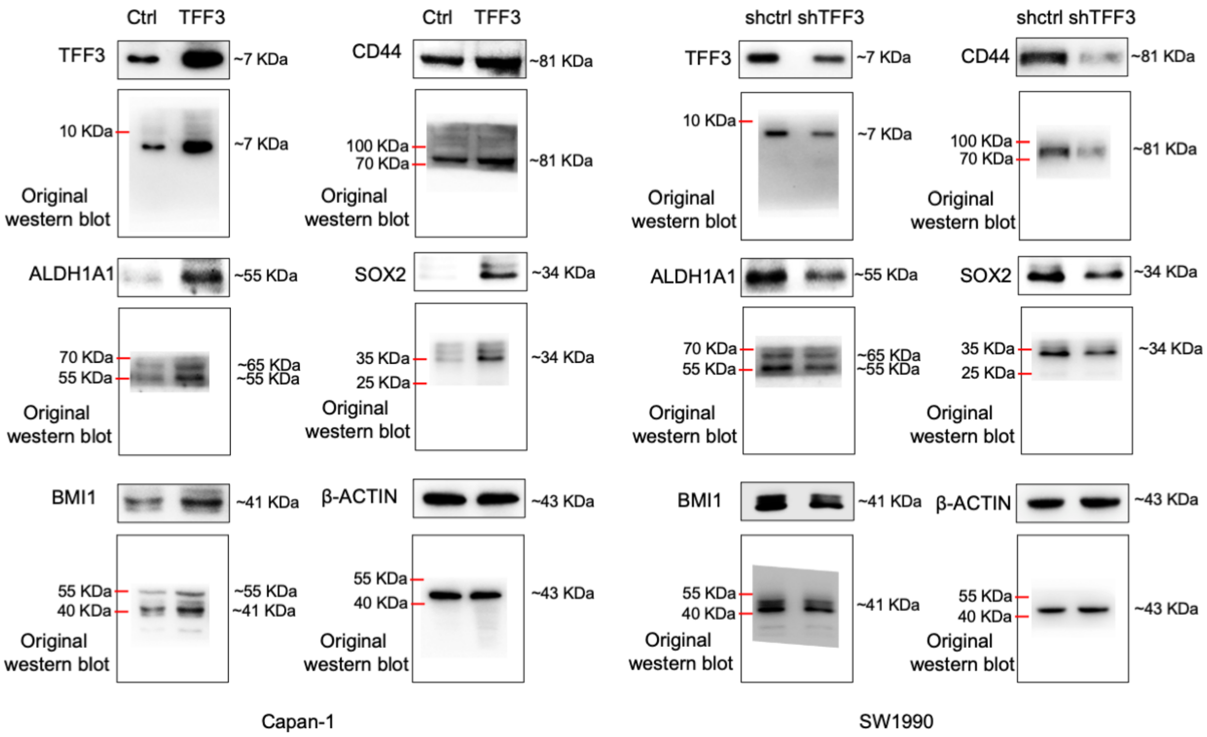

Fig. SO7 Original western blots for the western blot result in Fig. 6E

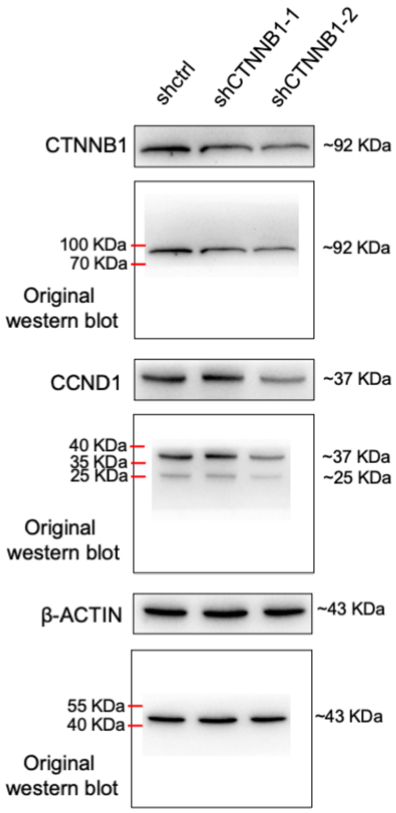

Fig. SO8 Original western blots for the western blot result in Fig. S6A

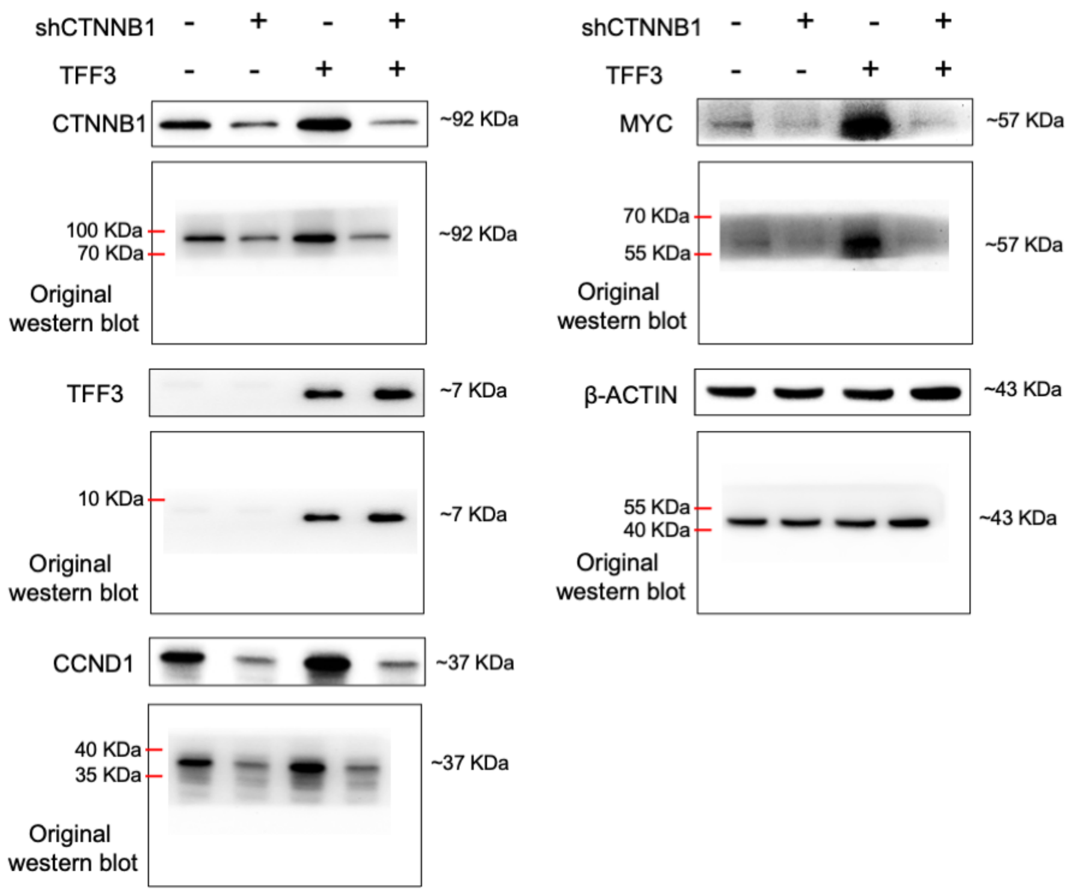

Fig. SO9 Original western blots for the western blot result in Fig. 7F
